# Trends in Clinical Trials: Investigating the Role of Inflammation in Alzheimer's Disease and Its Implications for Drug Development

**DOI:** 10.1002/alz70859_104762

**Published:** 2025-12-26

**Authors:** Raj Kumar, Rudra Pal, Sukhvir Kaur

**Affiliations:** ^1^ Chitkara University School of Pharmacy, Chitkara University, Baddi, Himachal Pradesh India

## Abstract

**Background:**

Emerging research highlights a significant role of inflammation in the pathogenesis of Alzheimer's disease (AD). This study aims to analyze trends in clinical trials, focusing on the pivotal role of inflammation in drug development and early‐stage AD, with the goal of identifying potential therapeutic targets for intervention.

**Method:**

This study analysis clinical reports on inflammatory markers in AD from the site ClinicalTrials.Gov with API on January 20, 2025, focusing on year‐wise clinical study statuses. This study includes an analysis of recruiting, completed, terminated, withdrawn and observation trials to identify trends in the elimination and addition of studies. It also includes a summary of study categories, outcomes, biomarkers such as IL‐6, TNF‐α, and CSF cytokines with their advanced techniques which are used to analyze them.

**Result:**

A total of 134 studies were analyzed, among which before the 2015 very few number of studies were found. However, after 2015 there was increase in number of studies, which indicates a growing interest among the researchers in role of the inflammation in AD. Among all the studies, 44 (33%) studies were completed, and 25 (19%) are still recruiting, indicating ongoing interest. Completed studies report the changes in inflammatory biomarkers which are correlating with disease progression. Seven studies (5%) were terminated and four studies (3%) were withdrawn due to recruitment challenges and/or inconclusive results. Additionally, 30 studies (22%) focused on peripheral inflammatory biomarkers. Before 2015, studies are found and focused mainly on biomarkers like IL‐6 and TNF‐α. While after 2015, studies focus shift to advanced imaging, such as PET scans with TSPO ligands. Advanced imaging and transcriptomic techniques have enhanced the understanding the role of inflammation in microglial and astrocytic cells, which offers new diagnostic and therapeutic possibilities.

**Conclusion:**

Research highlights the growing role of inflammation in AD, with a shift towards advanced imaging and molecular techniques. Despite challenges in study design and recruitment, significant progress has been made. Future research should combine imaging, molecular markers, and clinical interventions to effectively address inflammation in AD.

